# Classification for transmission electron microscope images from different amorphous states using persistent homology

**DOI:** 10.1093/jmicro/dfac008

**Published:** 2022-02-16

**Authors:** Fumihiko Uesugi, Masashi Ishii

**Affiliations:** Electron Microscopy Analysis Station, National Institute for Materials Science, 1-2-1 Sengen, Tsukuba, Ibaraki 305-0047, Japan; Materials Data Platform Center, 1-1 Namiki Tsukuba Ibaraki 305-0044, Japan

**Keywords:** amorphous structure, TEM image simulation, GaN, persistent homology

## Abstract

It is difficult to discriminate the amorphous state using a transmission electron microscope (TEM). We discriminated different amorphous states on TEM images using persistent homology, which is a mathematical analysis technique that employs the homology concept and focuses on ‘holes’. The structural models of the different amorphous states, that is, amorphous and liquid states, were created using classical molecular dynamic simulation. TEM images in several defocus conditions were simulated by the multi-slice method using the created amorphous and liquid states, and their persistent diagrams were calculated. Finally, logistic regression and support vector classification machine learning algorithms were applied for discrimination. Consequently, we found that the amorphous and liquid phases can be discriminated by more than 85%. Because the contrast of TEM images depends on sample thickness, focus, lens aberration, etc., radial distribution function cannot be classified; however, the persistent homology can discriminate different amorphous states in a wide focus range.

## Introduction

Interpreting amorphous images using transmission electron microscopy (TEM) is difficult owing to the lack of periodicity or symmetry. When analyzing a crystal structure with periodicity or symmetry using TEM, features can be extracted relatively easily using the Fourier transform. The contrast obtained in the TEM image significantly changes depending on the defocus, aberrations and sample thickness. In the analysis using the diffractogram obtained by a Fourier transform of the TEM images, the effects of these defocus and aberrations cannot be well distinguished. In the analysis of amorphous structures, the structure may be determined by performing a radial distribution function (RDF) analysis on the halo pattern obtained using selected area diffraction or nanobeam diffraction. To evaluate a small area, a nanobeam or angstrom beam that converges the electron beam is used; thus, the sample may be damaged, and it is unlikely that data showing the original structure can be obtained. Therefore, for material analysis, it is important to have a method that can identify regions with different sample states from TEM images with lower electron beam density and less damage to the sample than that of nanobeams.

Persistent homology (PH) is a concept of mathematical homology and is a data analysis method focusing on ‘holes’ [1,2]. Using PH, extracting information quantitatively in the form of data becomes possible. In PH, the circles are continuously enlarged from particular points scattered in the space. When the circles come into contact with each other and form a ‘hole’, the time of occurrence (birth time) and the time when the circles are further enlarged to form the inner ring increases. The time when it disappears is recorded as the death time. The graph exhibiting this is called a persistent diagram (PD). The PD represents the birth–death time as points scattered from the diagonal of the graph. Differences in the degree of dispersion of the starting points appeared in the PD. Its applications are being promoted in fields such as material science [3], molecular genetics and biochemistry. Algorithms suitable for machine learning (ML) using PD have also been developed, and Obayashi *et al.* developed and published them as HomCloud [4].

In the present work, we examined whether the TEM images of the amorphous state can be distinguished from images of liquids using PH. Because this is the first attempt, we considered the ideal state of a binary problem. Specifically, amorphous and liquid structures were created using classical molecular dynamics (MD) calculations. TEM simulation images were created using this output, and ML was performed using the PD obtained from the images. The samples were run on a binary GaN compound. Consequently, the accuracy was greater than 80%. It was found that PH is effective for identifying amorphous TEM images, such as amorphous and liquid phases. PH is often used for the analysis of three-dimensional data, such as the amorphous state, but in this study, we showed that it is also effective for two-dimensional data, such as TEM images.

## Data preparation

### Generation of amorphous and liquid structure using molecular dynamics simulation

Amorphous and liquid structure data for TEM simulation were generated using a classical MD simulation code, LAMMPS [5,6], which is distributed by Sandia National Laboratories. LAMMPS was operated via Pyiron [7,8], which is a Python library created by Janssen of Max Planck Institution and executed on a PC.

The present MD simulations adapted the whole direction periodic boundary condition and the isothermal–isobaric ensemble, which is suitable for structural transition, glass transition, crystallization, melting simulation, etc. The pressure and temperature of the model were kept constant under the isothermal–isobaric ensemble condition. Wurtzite GaN [9] crystals (29 × 24 × 10) were prepared as input data because an area of 50 Å × 50 Å× 50 Å was used for the TEM simulation. The Tersoff style [10] potential presented by Nord *et al.* [11] was adopted. The MD simulation for creating melting conditions was conducted under several temperatures between 1000 K and 6000 K. The calculation time was 10 000 steps (1 step = 1[fs]). Additionally, 5000 steps at 300 K to produce an amorphous structure (for quench treatment) were added to the above liquid conditions. The MD temperature is unrealistic, and the melting temperature is higher than the actual one [12,13].

**Fig. 1.**
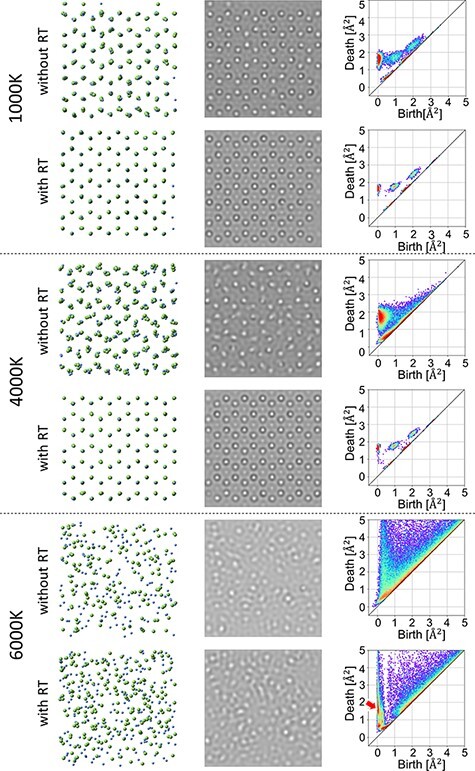
Results of molecular dynamics of 1000 K, 4000 K and 6000 K treatment. Perspective views (left column), TEM images (center) and PD (right) are shown. In the same temperature, the upper row is without RT treatment while the lower one is with RT treatment.

Perspective views of the 20 Å × 20 Å × 20 Å center region of the calculated structure in the [001] direction are shown in the left column of Fig. 1. It was found that the crystal structure and symmetry were maintained except for 6000 K, but as the temperature increased from 1000 to 4000 K, the structural disorder also increased. In structures treated with additional heat treatment at room temperature (RT), there was less disturbance and the crystal structure was almost normal. However, the symmetry of the structure treated at 6000 K was completely disturbed, which cannot be assumed to have a crystal structure. Furthermore, structures treated with additional heat treatment at RT could not recover the crystal structure. In the case of 6000 K, the structure in which additional RT treatment was added (or not) could not be distinguished from the former structure. Alternatively, the amorphous or liquid structures cannot be distinguished only from these perspective views.

### Generation of TEM simulation images

A commercial soft electron beam and image simulator (ELBIS) [14], which adopts the multi-slice method [15] and transmission cross coefficient (TCC) [16,17], was used to generate TEM simulation images. The input sample size for ML was 50 Å × 50 Å × 50 Å, and the simulated image size was 1024 × 1024 pixels (0.0488 Å/pixel) in nine defocus conditions as follows: −100, −50, −25, −13, 0, +13, +25, +50 and +100 Å. The defocus range of the present study is thought to be valid because the focus is adjustable in the defocus range in the case of Cs-corrected TEM; moreover, the TEM image contrast changes largely and inverses at defocus 0 Å. Small images for ML were cut into 256 × 256 pixels from the original simulated image. The slice thickness used to calculate the potential map is 1 Å. The Cs and Cc aberrations were −0.00050 mm and 1.37 mm, respectively. The other higher aberration coefficients were zero. The calculated images were saved in a 16-bit Tiff format. Because the Homcloud library uses 8 bit in grayscale, these images were transformed to an 8-bit format using the contrast limited adaptive histogram equalization (OpenCV) library.

One of the simulated images using former MD results is shown in the center column of Fig. 1. The image size was 20 Å × 20 Å in the [001] direction same as the perspective view. The trend of the simulated TEM image change with temperature was similar to that of perspective views. Regardless of RT treatment, TEM images from MD results treated at 1000 K and 4000 K had a small disturbance but preserved the symmetry of the pristine crystal structure. However, at 6000 K treatment, it was found that the symmetry disappeared regardless of RT treatment. They had a crystal structure and were believed to be amorphous and liquid states.

### Persistent diagram (PD) from MD 3D data

PDs were generated using the HomCloud [4] library of Python. The input data were former MD results of Ga and N radii calculated using values obtained by Ishimaru *et al.* [18] and were 1.5625 Å and 0.49 Å, respectively. PDs from the 3D structure data are shown in the right column of Fig. 1. In PDs from structure data without RT treatment, it was found that localized birth–death pairs were dispersed as the temperature increased and each birth–death pair peak disappeared at 6000 K. In the data for the 1000 K + RT and 4000 K + RT data, peaks appear clearly, but in the case of 6000 K + RT, peaks do not appear. The situation shown by the PD at each temperature is consistent with the perspective view and TEM image. The peaks near (birth, death) = (1,1.7) and (2,2.4) were due to the existence of a surface in the structural model used in this study and did not appear in an infinitely large model.

**Fig. 2.**
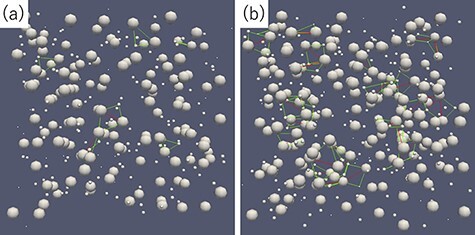
Atomic arrangements of liquid (a) and amorphous (b). Optimal volumes corresponding to the peak position indicated by red arrow in the right bottom PD of Fig. 1 are indicated with green and red lines. For visibility, atomic radii are made smaller than the calculated value.

Figure 2 represents the optimal volumes of the peak position indicated by the red arrow in the bottom-right PD of Fig. 1. The optimal volumes are represented by the green and red lines, respectively. It can be observed that there are more atomic arrangements satisfied with the optimal volume in Fig. 2b than in Fig. 2a.

The state of 6000 K + RT was relaxed by changing the liquid state to RT and had a state different from the liquid state. This state was defined as an amorphous state because it was clearly different from the liquid state when compared by PD. Hereafter, data in 6000 K treatment with/without RT was used as an amorphous state and liquid in common. Here, 6000 K + RT was not intended to reproduce quenching. The combination can be regarded as an amorphous structure resulting from pure structural stabilization by relaxation from a random atomic arrangement. In other words, it is considered to be the most stable structure determined by the atomic potential, independent of the quenching rate. Similarly, the simulation of 6000 K without RT did not assume difficult ultra-high temperature TEM observations but rather an ideal random atomic arrangement, perhaps obtained by ion implantation.

## Examination by each identification method

### Annular averaged diffraction intensity (AADI)

**Fig. 3.**
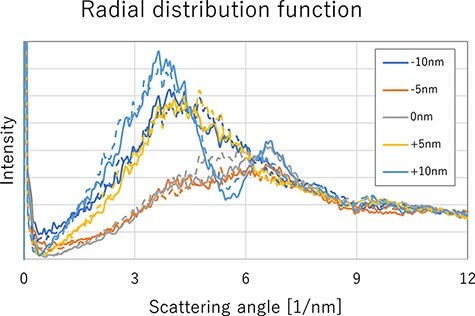
AADI of simulated TEM images in various defocus. Solid lines are liquid (heat treatment without RT), and dashed lines are amorphous (with RT).

We attempted to classify the states using the AADIs. AADI is obtained by averaging Fourier-transformed TEM image in the radial direction and is approximately equivalent to the radial distribution in reciprocal lattice space. AADIs from simulated images of various foci are shown in Fig. 3. The abscissa in Fig. 3 is labeled as scattering angle, which can be considered as distance in the RDF, although it is the reciprocal. The profiles changed in a complicated manner depending on their defocus, regardless of whether they were amorphous or liquid. Therefore, it was difficult to determine whether the image was taken from an RT-treated sample or not.

### ML using persistent homology

As a present ML method for PH, we applied Obayashi’s method [19], which uses persistent images (PI) [20] for learning objects. The transformation from PD to PI was performed using the method described by Adams *et al.* [20]. In the PI, each birth–death point is assigned a weight depending on the distance from the diagonal of the PD. The larger the distance, the larger the weight.

Because the learning method is detailed in [19], only a simple procedure is shown below.

**Fig. 4.**
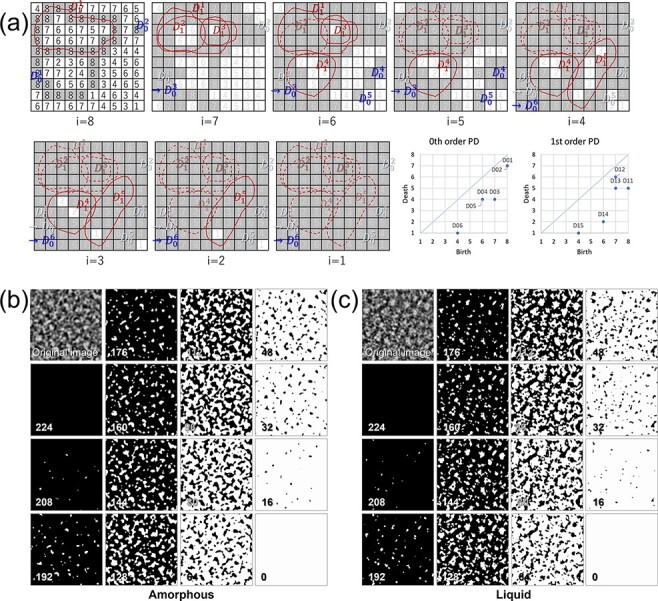
Filtration of cubical set using image intensity. (a) Example using a small set. Numbers in cells are assumed to be image intensity. Changing the intensity (i) from high to low, expand the areas. Zeroth simplices (i.e. islands = connected components) and first ones (i.e. rings) are born or die depending on the intensity. Blue and red indexes represent zeroth- and first-order simplices, respectively. The zeroth- and first-order PD are shown in the bottom right of (a). When the islands come into contact with each other, they die, and another island is born. (At *i* = 7, islands D_0_^1^ and D_0_^2^ died on contact with each other and D_0_^3^ was born.) (b) and (c) show amorphous and liquid filtration. White areas expand depending on the intensity. (The image size is 12.5 Å × 12.5 Å. Numbers indicate intensity lower limit for filtration.).

1. The zeroth- and first-order PDs were calculated using simulated TEM images. Since the filtrations were done from high to low intensity, the points that represent birth–death pairs are displayed under the diagonal in the PD. 0th order simplices are connected components, and the first ones are rings. An example is shown in Fig. 4a.
2. Both PIs are transformed from PDs.
3. Logistic regression and support vector classification for two-class classification with cross validation were conducted using zeroth- and first-order PIs separately or in combination. The PI data were divided into 75% for training and 25% for testing for the cross validation.

The results of filtration on amorphous and liquid TEM images are shown in Fig. 4b and c, respectively. In the case of the amorphous results in Fig. 4b, it seems that the changing manner of the white area is rather gradual and individual components grow larger than the liquid ones. However, in the case of liquid, smaller islands are born and die more frequently, and they have finer structures than amorphous ones. According to examples (b) and (c), it seems that the arrangement of atoms in amorphous has a certain structure, while it is more random in liquids than in amorphous.

**Fig. 5.**
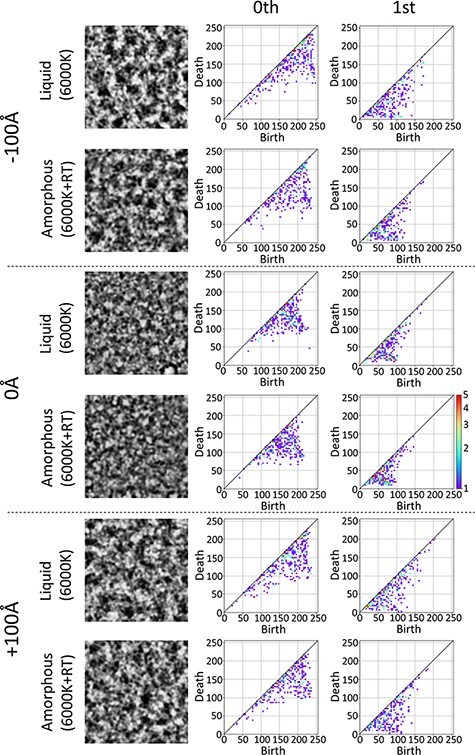
TEM images and PDs of zeroth- and first-order.

Figure 5 shows a part of the TEM image and the PD used for ML. According to the results of the previous filtration, there seems to be a difference in the way islands and rings are formed and disappear between amorphous and liquid; however, because of the focus dependence of the image, it is not clear at a glance where the characteristics of PD exist. Because the actual data is slightly out of focus, it is necessary to obtain the features using the data of many focus sets. ML is effective for this purpose, and it was executed by combining the scikit-learn and Homcloud [4] mentioned above. The Homcloud [4] library was used to create PD and PI from TEM images, while the scikit-learn library [21–22] was used for ML using PIs. The logistic regression (LR) and support vector classification (SV) results were compared. MLs were conducted using each order PI separately or using a combined zeroth-order and first-order PI. This learning method is a supervised learning, in which all amorphous and liquid images were labeled as 0 or 1. In both methods, the regularization parameters were 0.01, and the hinge function was used in SV. Accuracy was defined as the ratio between the correct number and the total image number.

For supervised learning insensitive to focusing, the amorphous and liquid phases can be discriminated by 0.773 with LR and 0.817 with SV. In the case of learning the zeroth-order PIs and first-order ones separately, the zeroth and first accuracies were 0.649 and 0.756 for LR and 0.672 and 0.790 for SV, respectively.

**Fig. 6.**
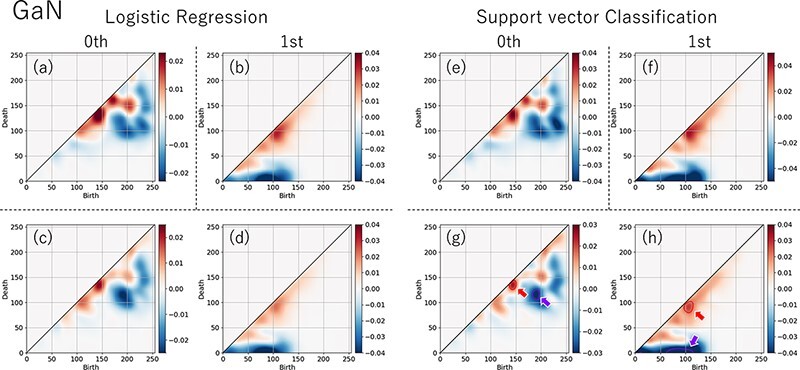
Visualized learning results using PD. (a)–(d) are results of logistic regression and (e)–(h) support vector classification. The upper rows show results using zeroth- and first-order PI data separately. The lower rows combined zeroth- and first-order PI data.

The training results for GaN are presented in Fig. 6. The blue and red areas represent the features inherent to amorphous and liquid phases, respectively. Both LR and SV classify PI by substituting the value of the function $h\left( {\bf{x}} \right)$ to Sigmoid function, where the vector ${\bf{x}}$ is the vectorized PI. Specifically, $h\left( {\bf{x}} \right)$ is obtained by operating the input vector ${\bf{x}}$ with the weight vector ${\bf{w}}$ that is obtained using ML.

$h\left( {\bf{x}} \right) = {\bf{w}} \cdot {\bf{x}}$ (omitted constant for simplicity)

Substituting $h\left( {\bf{x}} \right)$ to Sigmoid function, makes it correspond to the label 0 for amorphous and 1 for liquid.

If $h\left( {\bf{x}} \right) \lt 0$, then Sigmoid function $\mathbin{\lower.3ex\hbox{$\buildrel\gt\over{\smash{\scriptstyle=}\vphantom{_x}}$}}$0.5, and ML predicts the label 0. If not, Sigmoid function $\mathbin{\lower.3ex\hbox{$\buildrel\lt\over
{\smash{\scriptstyle=}\vphantom{_x}}$}}$0.5, and it predicts the label 1. Each point of PI is a one-dimensional vector with a nonnegative value. Each element of ${\bf{w}}$ represents the contribution of each element of ${\bf{x}}$. Since ${\bf{x}}$ is a nonnegative vector, each element of weight ${\bf{w}}$ must be minus (plus) for amorphous (liquid). Figure 6 is drawn with the weight ${\bf{w}}$ . These areas allow the machine to determine the difference between both phases. The upper row shows the classification results using the zeroth- and first-order PI data separately ((a) and (e) for zeroth and (b) and (f) for first). The lower row (c), (d), (g) and (h) show the results using the combined zeroth- and first-order PI data. Note that even when the zeroth- and first-order data are combined, the features of zeroth and first are obtained separately, as shown in the lower row. From these figures, it can be observed that the blue areas are farther away from the diagonal than the red areas in both the zeroth-order and first-order PDs. Since points in a PD represent lifetimes (= death time−birth time) of simplices, a long distance from the diagonal means a long lifetime i.e. the longer the distance from the diagonal, the larger is the structure. Such a clear difference favors the use of PDs to detect the difference between amorphous and liquid images. As described above, when the zeroth- and first-order data were trained separately, the ability to discriminate between amorphous and liquid was approximately 65% and 75%, respectively, indicating that the contribution to discrimination ability was higher for the first-order training results. This suggests that the structure of the ‘ring’ in amorphous materials, as discussed in Fig. 4, is effective for discrimination. Although it is difficult to discriminate amorphous and liquid phases using only the zeroth-order PD, which does not reflect the short-range structural order, the ability to discriminate each phase is enhanced when combined with the first-order PD. This finding indicates that the ability to discriminate can be further improved by incorporating the rings as well as the formation and disappearance of their connecting components. Therefore, in the case of amorphous materials, it can be considered that the connecting components are formed at the initial state of ring generation.

**Fig. 7.**
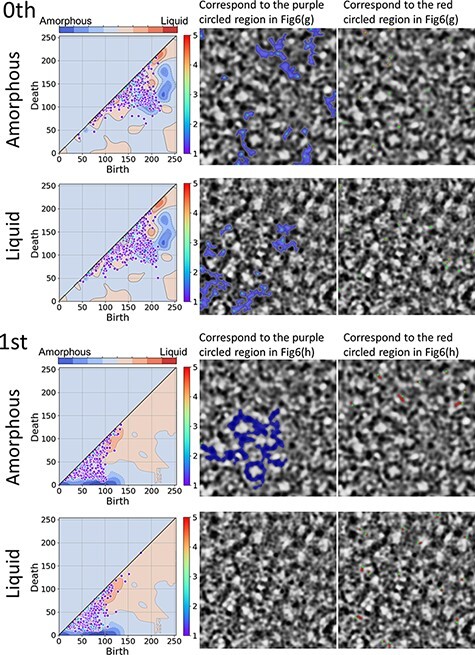
Inverse analysis result using just-focused amorphous and liquid TEM image. The areas of the inverse analysis from the selected birth–death pairs of amorphous are shown in the first and third rows; those of liquid are shown in the second and fourth rows. The zeroth and first PDs are shown in the left column. The blue (red) areas on the TEM image in the second (third) column are from the birth–death pairs in the amorphous (liquid) superior area in Fig. 6g and h. The green points are the birth points.

Figure 7 illustrates the inverse analysis results from Fig. 6., in which amorphous and liquid TEM images of just focus are used as examples. Inverse analysis represents the pattern in a TEM image that a particular region of PD corresponds to. The upper two rows show the zeroth-order results, and the lower two rows show the first-order ones. In each result, upper (lower) shows amorphous (liquid) results. The left column shows the PD superimposed on the contour map converted from the results in Fig. 6. The second and third columns show inverse analysis results and blue (red) patterns corresponding to purple (red) circled region in Fig. 6g and h. The purple (red) circled regions are amorphous (liquid) superior ones in the PD. The green points are their birth points. In the zeroth-order results, there was only a slight difference between amorphous and liquid in terms of the number of regions corresponding to the regions surrounded by the purple and red lines. In the first-order results, the pattern corresponding to the area surrounded by the purple (red) line was more in Amorphous (Liquid) than in Liquid (Amorphous). Although the correct answer ratio learned using only the zeroth-order PD is lower than using only the first-order PD as mentioned above, the inverse analysis results are in agreement with it.

**Fig. 8.**
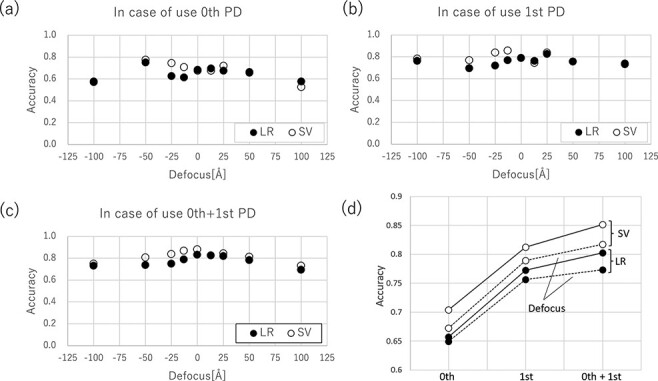
Accuracy of amorphous and liquid TEM image using logistic regression or support vector classification for (a) zeroth-order, (b) first-order, and (c) a combination of zeroth- and first-orders.

Figure 8 shows the dependence of the accuracy on the amount of defocus for LR and SV. Figure 8 shows the predicted accuracy using (a) only the zeroth-, (b) first- and (c) zeroth- and first-order PDs. The average accuracy of each of these (a)–(c) is summarized by the dashed line in Fig. 8d. In these figures, the closed and open circles represent predictions using LR and SV, respectively. From Fig. 8d, it can be observed that SV is superior to LR. Furthermore, the improvement in accuracy discussed above, zeroth-order < first-order < zeroth- and first-order (denoted by ‘0th + 1st’ in this figure), is graphically summarized.

Importantly, we found that the prediction accuracy is different when the defocus region in Fig. 8a–c is limited to ±25 Å (in-focus) from the results when the defocus region is considered to be up to ±100 Å (defocus). The results of the in-focus are summarized as solid lines in Fig. 8d. From a comparison between the solid and dashed lines in this figure, the accuracy of the in-focus is higher than that of the defocus. Considering this result from a crystallographic point of view, it may correspond to the fact that the contrast transfer function differences are not large around the focus, to ensure that the structural information is easily reflected in the image, whereas in the case of defocus, the structural information is lost due to the overlapping interference fringes. Considering it from the perspective of ML, it indicates that the structural information is consequently reflected in the features of Fig. 6, even though the model does not contain any prior information about the structure.

Finally, we reconsidered the accuracy from a practical point of view. In this case, we discuss a relatively large defocus amount (±100 Å). However, in recent TEMs, it is possible to adjust the focus within ±25 Å using a Fourier transform during operation. The solid line presented in Fig. 8d shows that when the amount of defocus is within ±25 Å, the accuracy improves to 0.803 for LR and 0.851 for SV. Therefore, we may identify the amorphous state with a high discrimination accuracy of more than 85%, even in normal operation.

## Concluding remarks

We studied the effectiveness of the PH to classify the TEM images from two different amorphous states: liquid and amorphous. After transforming PI from PD using simulated TEM images of each state with different defocus, two ML methods, LR and SV, were applied to discriminate them. Consequently, in the case of GaN, the accuracy was over 85% in the SV for defocus between −25 Å and 25 Å. PH is known to be effective for three-dimensional data, but it has also been found to be effective even in less dimensional data, such as two-dimensional image data. However, the three-dimensional structure/information, which changes in a complicated manner owing to the difference depending on the defocus in TEM, still exists. Two-dimensional PD is created using the pixel intensity of the image. It is not possible to simply associate a PD created from three-dimensional structural data with one created from two-dimensional image data on a one-to-one basis. Therefore, PH also has a difficult phase in interpretation. Although not limited to TEM images, actual measurement data contain noise. As PH is believed to be vulnerable to noise, its application to TEM images might not be suitable. However, a low-pass filter is often applied to TEM images to avoid dropping information, thus noise is not considered a big problem for PH applications. In the present work, we considered only the change in defocus, but PH can be a new tool for analyzing amorphous TEM images.
